# The first report of *Nervilia lilacea* Jum. & H. Perrier (Orchidaceae, Epidendroideae) from Kenya and the Northern Hemisphere

**DOI:** 10.3897/phytokeys.135.46629

**Published:** 2019-11-11

**Authors:** Jing Tian, Vivian Kathambi, Peris Kamau, Geoffrey Mwachala, Itambo Malombe, Guang-Wan Hu

**Affiliations:** 1 CAS Key Laboratory of Plant Germplasm Enhancement and Specialty Agriculture, Wuhan Botanical Garden, Chinese Academy of Sciences, Wuhan 430074, Hubei, China Wuhan Botanical Garden, Chinese Academy of Sciences Wuhan China; 2 Sino-Africa Joint Research Center, Chinese Academy of Sciences, Wuhan 430074, Hubei, China Sino-Africa Joint Research Center, Chinese Academy of Sciences Wuhan China; 3 University of Chinese Academy of Sciences, Beijing 100049, China University of Chinese Academy of Sciences Beijing China; 4 East African Herbarium, National Museums of Kenya, P.O. Box 451660-0100, Nairobi, Kenya National Museums of Kenya Nairobi Kenya

**Keywords:** Flora of Kenya, illustration of *Nervilia lilacea*, new record, Orchidaceae, taxonomy

## Abstract

*Nervilia lilacea* is recorded from Kenya as well as the Northern Hemisphere for the first time. A plate of ink drawing and a distribution map are provided based on the new collection.

## Introduction

*Nervilia* Commerson ex Gaudichaud-Beaupré in Freycinet (1829: 421) comprises ca. 80 species, distributed from tropical, subtropical and warm temperate regions of Africa, Asia, Australia, and the Southwest Pacific islands (Govaerts et al. 2019). There are 15 species of *Nervilia* recorded in Africa (Govaerts et al. 2019). Among these species, five of them have been recorded in Kenya (Petersson 1990, 1991; Stewart and Campbell 1996; Olszewski 2004; Nusbaumer et al. 2011; Govaerts et al. 2019). In April 2018, during a field survey in Nandi Forest, *N. lilacea* Jumelle & Perrier (1912: 197) was collected from Kenya (the northern side of the equator) for the first time. We report it here with a plate of ink drawing and a distribution map based on the new collection.

## New record

### Nervilia lilacea

Taxon classificationPlantaeAsparagalesOrchidaceae

Jum. & H.Perrier, Ann. Fac. Sci. Marseille 21(2): 197, 1912

956CD190-51DE-5FA5-BCBA-FD775E06597C

Fig. 1

= Nervilia gassneri Börge Pett. in Nord. J. Bot. 9: 492. 1990. Type. Malawi: Southern Prov., Zomba Distr., Zomba Plateau, 1530 m, 15 July 1984. *Petersson and Gassner 359* (holotype: UPS, image seen!; isotype: BP, K, image seen!, LISC, LMU, MAL, NHT, SRGH).

#### Type.

Madagascar: Centre, massif de Manonarivo, bois humides, 1000 m, fl., *Perrier de la Bâthie 1873* (holotype: P [P00094725], image seen!).

**Figure 1.**
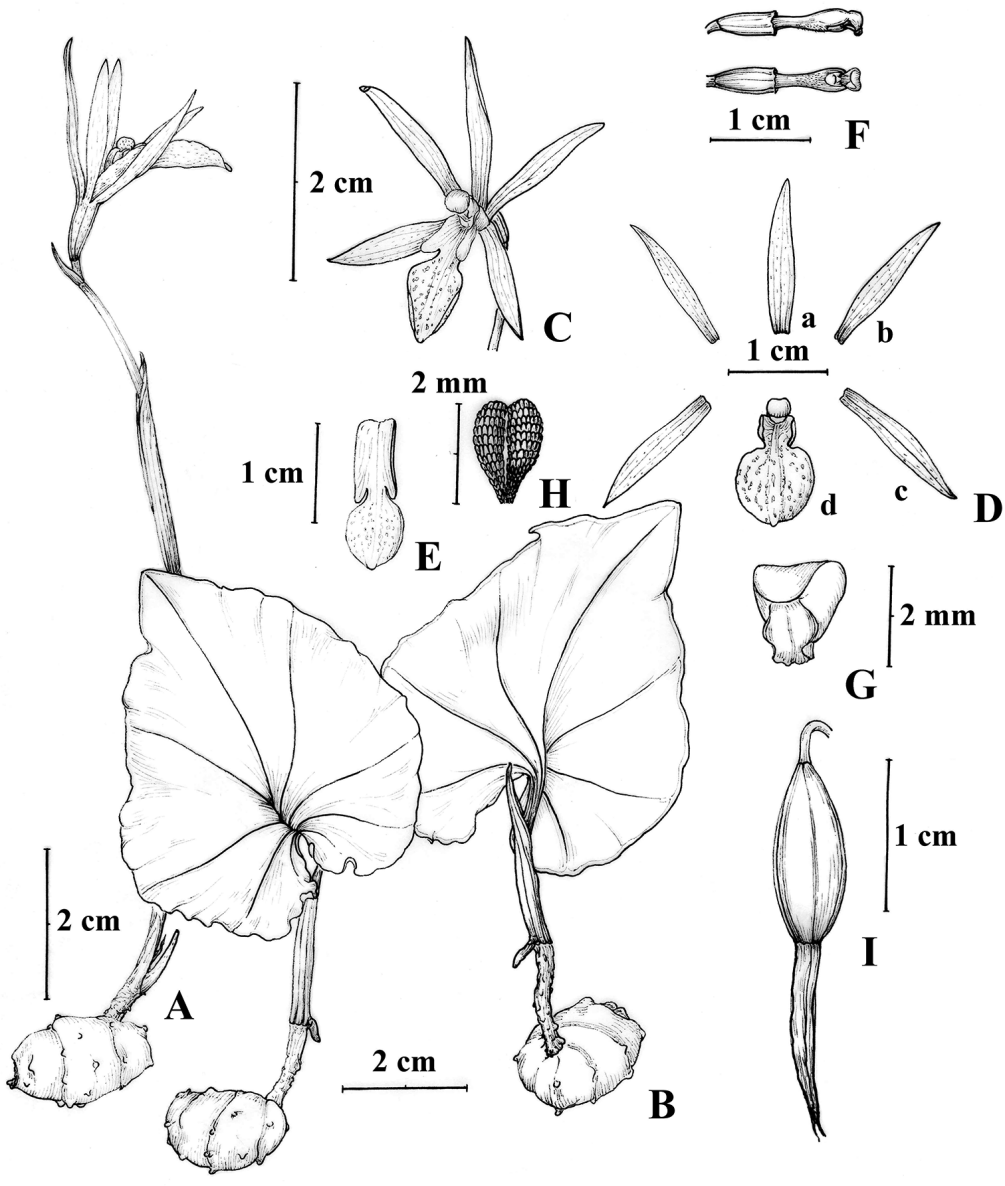
*Nervilia lilacea***A** plant with flower **B** plant with leaf (adaxial and abaxial leaf) **C** flower, frontal view **D** floral pieces dissected (**a** dorsal sepal **b** lateral petal **c** lateral sepal **d** lip) **E** lip **F** column, lateral and ventral view **G** anther **H** pollinia **I** capsule (Jing Tian drew it from *FOKP-1530* specimen).

#### Specimens examined.

Kenya. Nandi North District, Spetonok, 0°22'32"N, 35°00'29"E, elevation 2000 m, 22 April 2018, *FOKP-1530* (EA, HIB).

#### Distribution.

**Figure 2.**
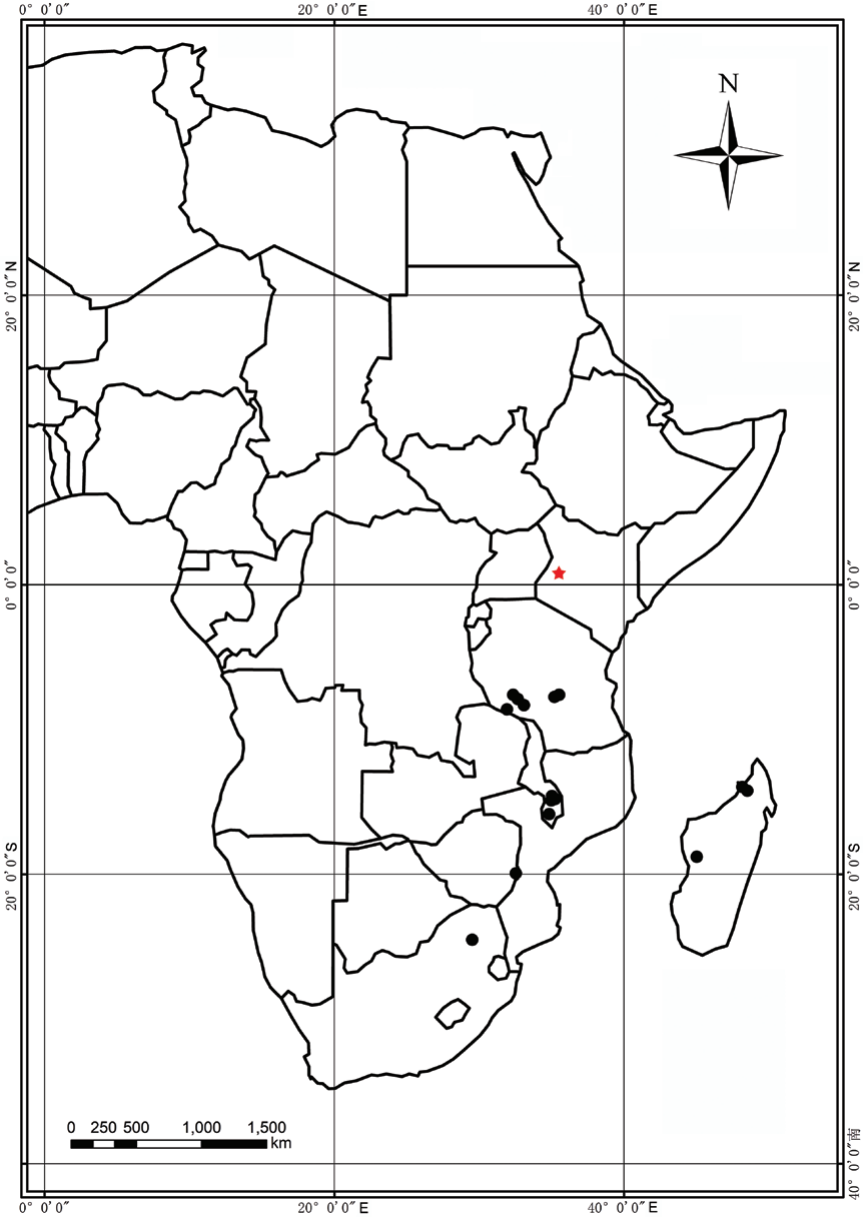
Distribution map of *Nervilia lilacea*, with the new collection *FOKP-1530* shown by a red star [other points re-drawn after Petersson (1990, 1991) and specimen records]

Madagascar, Malawi, South Africa, Tanzania, Zambia, Zimbabwe, Kenya (new record). (Fig. 2)

#### Habitat and phenology.

Tropical rain forest floor margins at elevation 200–2000 m a.s.l.. Flowering from March to April was observed in Kenya.

## Supplementary Material

XML Treatment for Nervilia lilacea
